# Associations between prenatal serum levels of leptin, IGF-I, and estradiol and adolescent mothers’ height gain during and after pregnancy

**DOI:** 10.1371/journal.pone.0228706

**Published:** 2020-02-11

**Authors:** Reyna Sámano, Hugo Martínez-Rojano, Gabriela Chico-Barba, María Hernández-Trejo, Raymundo Guzmán, Gabriel Arteaga-Troncoso, Mariana Alejandra Figueroa-Pérez, Rosa María Morales, Gabriela Martínez

**Affiliations:** 1 Departamento de Nutrición y Bioprogramación, Instituto Nacional de Perinatología, Mexico City, Mexico; 2 Sección de Posgrado e Investigación, Escuela Superior de Medicina del Instituto Politécnico Nacional, Mexico City, Mexico; 3 Escuela de Enfermería, Facultad de Ciencias de la Salud, Universidad Panamericana, Mexico City, Mexico; 4 Neurobiología del Desarrollo, Instituto Nacional de Perinatología, Mexico City, Mexico; 5 Departamento de Diagnóstico por Imagen, Instituto Nacional de Perinatología, Mexico City, Mexico; 6 Departamento de Fisiología y Desarrollo Celular, Instituto Nacional de Perinatología, Mexico City, Mexico; 7 Universidad Iberoamericana, Mexico City, Mexico; 8 Coordinación de Nutrición., Universidad del Valle de Mexico-Chapultepec, Mexico City, Mexico; Texas A&M University College Station, UNITED STATES

## Abstract

Mexico is within the top three Latin American countries with the highest proportion of adolescent pregnancies while being in the lowest ten Latin American countries in terms of height. It is still unclear how much growth in adolescence is affected by pregnancy; therefore, this study was designed to study the association between prenatal serum concentrations of leptin, IGF-I, and estradiol and the increase in the height of a group of pregnant adolescents between the 28^th^ week of gestation to one year postpartum. We conducted a cohort study from 2009 to 2017 in pregnant adolescents in their third trimester of pregnancy receiving prenatal care at Mexico’s National Institute of Perinatology. Data on hormones, other covariates, and confounding variables were analyzed through bivariate analysis and then a linear univariate analysis. Our patients were an average of 15.5±1 years and gained an average of 9.5 mm during the study period. A Pearson’s correlation showed a positive and significant height increase between height and leptin and IGF-I, and negative between height and estradiol. The general linear model (adjusted by age, bone age, gynecological age, parent’s stature, breastfeeding, body fat, energy intake, and BMI) found that leptin and estradiol serum concentrations explained 39.6% of height increase; IGF-I did not have any predictive effect. Leptin and estradiol concentrations in the third trimester of pregnancy are associated with increased height in our group of teenage mothers. No effect association was observed between height and IGF-I concentrations.

## Introduction

Teenage pregnancies have been reduced in developing countries at lower rates than in developed countries. While developed countries managed to decrease adolescent births by 41.9% between 1995 and 2015, developing countries decreased adolescent births by 26.5%. More specifically, while Latin American and Caribbean countries managed to reduce adolescent births by 15.3%, in Mexico, the reduction was only 12.9% [1]. Adolescent pregnancies are a public health problem because of the negative consequences these pregnancies can have on maternal health [2]. Adolescent mothers have an increased risk of preeclampsia [3], inadequate gestational weight gain, [4], and height stunting [5] due to a nutrient competition between the mother and the fetus [6,7]. Maternal growth retardation has, in turn, been associated with adverse maternal and neonatal outcomes [8].

Several genetic, hormonal, and environmental factors come together to explain height growth in adolescents. Leptin is a hormone involved in growth and weight retention during the postpartum period, [9] and estrogens play a role in bone growth and maturation of the growth plates. In the course of bone growth, estrogen is needed to close epiphyseal growth plates in women and men. Estrogens act by inhibiting the differentiation of osteoclasts [10], meaning that estrogen can affect bone age and expected height. Longitudinal bone growth is mediated by growth hormone (GH), insulin-like growth factor I (IGF-I), and more importantly, local IGF-I in the growth plate. IGF-I regulates cell proliferation; as it is synthesized primarily in the liver [11], IGF-I concentrations reflect GH secretion [12]. Puberty produces significant increases in IGF-I [13] as a result of the growth hormone increase mediated by sex hormones [14].

It is still unknown exactly how much growth in adolescence is affected by pregnancy. Our research group has observed—in person and the literature—contradictory results in Mexican populations in recent years. In 2006 we saw that teenage girls who gave birth to healthy babies did not gain height during pregnancy [15]. A few years later, we observed an average growth of 15 mm in a group of teenage mothers during their first year postpartum [16]. An external study by Pauli et al. in adult Mexican-American women did not find statistically significant differences in stature between those who had their first child before age 18 and those who gave birth after 18 (mean difference: 3.7 mm CI 95%; -27.9, 35.4) [17]. Factors that could modulate growth during adolescent pregnancy include energy intake, the number of pregnancies [18], and expected height in relation to the height of the adolescent’s parents [5]. Despite needing increased calorie intake during pregnancy, many pregnant women do not meet the recommended daily intake [19]. These findings, taken together, sparked a renewed interest in measuring height in adolescent mothers and the biochemical mechanisms associated with this process.

We, therefore, proposed a study to understand better the factors associated with adolescent height gain during pregnancy. We believe that fetal competition for nutrients is a factor in growth retardation or interruption in adolescents and that this modifies mothers’ physical development [8,20–23]. The purpose of this study was to assess whether prenatal serum concentrations of leptin, IGF-1, and estradiol were associated with the increase in the height of a group of pregnant teenage women, measured from the 28^th^ week of gestation to one year postpartum. The World Health Organization (WHO) has established an average height gain for girls aged 12 to 18 years of 15 mm per year, with rates ranging from 43 mm/year at 12 years to 1.0 mm/year at 17 years [24]. Our null hypothesis is that our population of pregnant adolescents will not show statistically different rates than these average growth rates for adolescents.

## Material and methods

### Study design

We conducted a cohort study from 2009 to 2017, where participants were pregnant adolescents in their third trimester of pregnancy receiving prenatal care at Mexico’s National Institute of Perinatology (*Instituto Nacional de Perinatología* or INPer). INPer is a public tertiary care institution that treats high-risk pregnancies, including pregnant adolescents who do not have formal employment or health insurance. The participants were residents of Mexico City and surrounding states. All participants were adolescents as defined by the WHO: as a person between 10 and 19 years. However, in our sample, we did not have any participants younger than 11 nor older than 17 years old.

A sample size was calculated to be representative of the INPer’s typical population of pregnant adolescents over three years (700). The sample size was calculated to compare two means for a finite population with a 95% confidence interval and a power of 80%. The total calculation of the sample was 151 participants, and an additional 25% was added to account for abandonment (n = 190).

Sampling was non-probabilistic, and all patients who met inclusion and exclusion criteria were included in consecutive fashion. Inclusion criteria included: being an adolescent in the third trimester of pregnancy; not having any evidence of chronic, infectious, or metabolic diseases in the participant’s medical records; bearing a healthy singleton pregnancy; and receiving care during childbirth from INPer’s obstetrics department. Exclusion criteria included: smoking or use of illicit drugs and carrying a pregnancy resulting from rape. Participants who developed pregnancy-induced hypertension or gestational diabetes were eliminated to avoid any bias.

Potential participants were recruited between 2009 and 2016 by INPer nutritionists during outpatient consultations. After having the study purpose, risks, and benefits explained in plain-language terms, all participants signed informed assent, declaring that they were participating out of their own free will. Their respective parents or guardians signed informed consent. Fifteen more patients than necessary were recruited, meaning that two hundred and five teenagers began the study; however, 37 participants were lost-to-follow-up and subsequently eliminated from the study. The total sample was, therefore, 168 pregnant adolescents.

**Fig 1:**
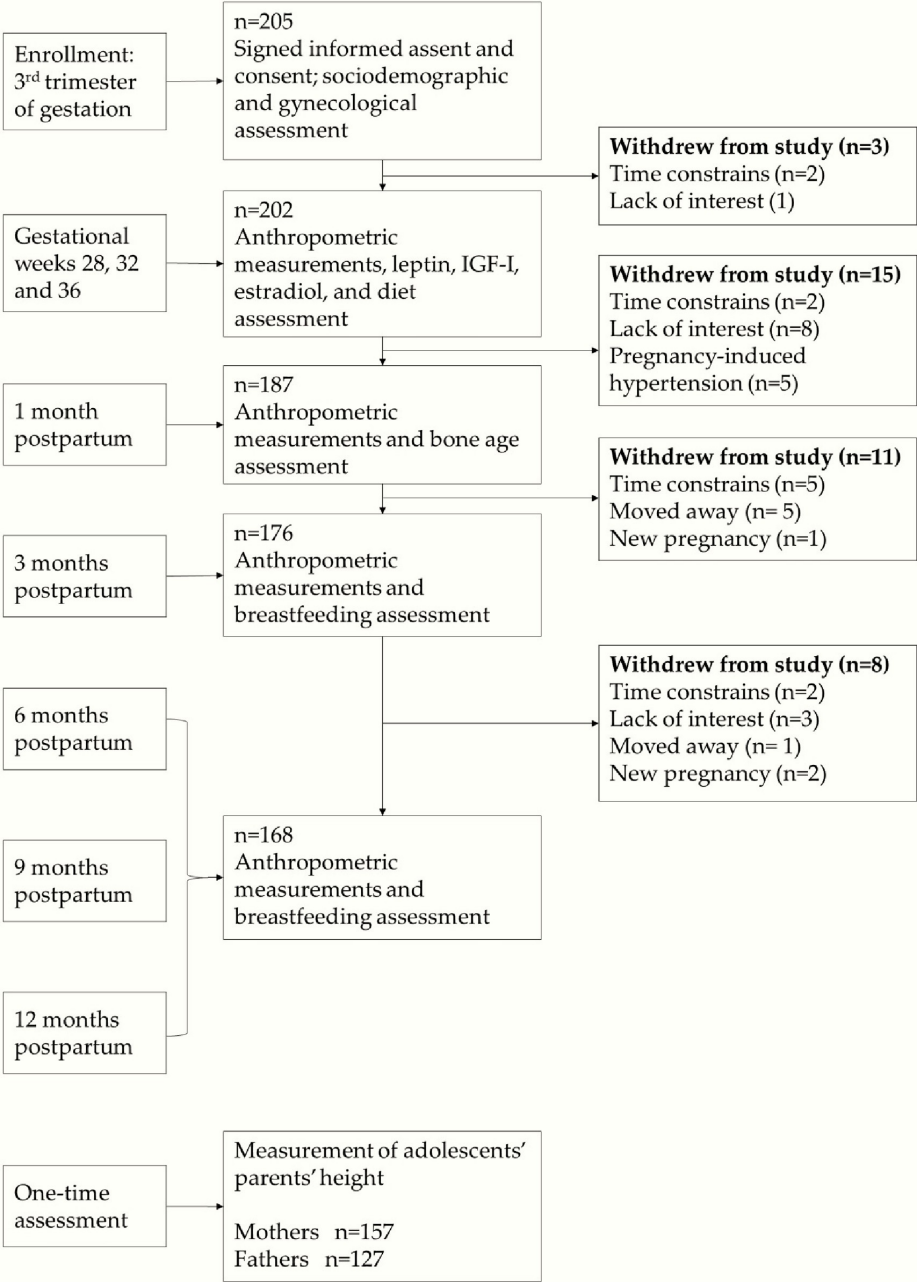
Participant flow chart.

Per INPer protocol, all participants had their first assessment prior to their third trimester and were therefore recruited into the study before gestational week 28. Study visits during pregnancy occurred at gestational weeks 28, 32, and 36. Postpartum study follow-up visits occurred at months 1, 3, 6, 9, and 12. (Fig 1) shows the assessments performed at each visit and how many participants were lost at each step of the study. To identify possible selection bias, sociodemographic variables, clinical characteristics, and leptin, IGF-I, and estradiol concentrations were compared between those who completed the study and those who withdrew. No statistically significant differences were identified between groups, confirming the absence of any apparent selection bias.

### Follow-up procedures

#### Evaluation of diet and breastfeeding

Energy and macronutrient intake was assessed at the first visit (28^th^ week of gestation) using a food frequency questionnaire [25]. The number of calories ingested daily was compared (as a percentage) against the values proposed by the Institute of Medicine [26]. The distribution of energy into macronutrients—percent of carbohydrates, proteins, and fats—was also calculated.

During follow-up visits at months 3, 6, 9, and 12 months postpartum, the participating mothers were asked if they were feeding their child exclusively with breast milk, mixed (breast milk and formula), or exclusively with formula. The duration of exclusive breastfeeding was defined as the time in months that the baby was only fed with breast milk [27].

#### Anthropometric assessments

Trained health professionals performed anthropometric measurements using the Lohman technique [28]. Pregestational weight was self-reported; participants were asked to report their weight three months before pregnancy. This pregestational weight—in kilograms—was divided by the height recorded during the first trimester—in meters squared—to provide the pre-pregnancy BMI (weight/height^2^). This index was classified in percentiles according to WHO growth charts for sex and age [29]. From week 28 of gestation to delivery, all participants were weighed every four weeks. Measurements were always taken between 7 and 10 AM, after an eight-hour fast, using a digital scale (SECA model 370, with precision 0.100 kg). Gestational weight gain was defined as maternal weight at the end of pregnancy minus pregestational weight.

*Height and height increase*. Height was measured every three months starting from the 28^th^ week of gestation up to 12 months postpartum, using a stadiometer (SECA model 274, with precision 0.10 cm). Height was classified in percentiles according to the WHO growth charts by sex and age [29]. Height gain was calculated by subtracting the participant’s height at 12 months postpartum from the participant’s height at 28 weeks of gestation. This height increase was divided into tertiles, where the tertile I was a minor increase (≤7 mm), tertile II was an increase between 7 to 11.9 mm, and tertile III was an increase ≥12 mm.

*Bone and gynecological age*. Participants’ bone age was evaluated 30 days postpartum with an x-ray of the non-dominant hand-wrist. The epiphyseal ossification centers were evaluated using the Greulich and Pyle atlas [30]—images of this atlas have shown a high correlation with digital techniques [31]. Bone age was classified as below, equal to, or above chronological age; the frequencies of these categories were compared to tertiles of height increase. The gynecological age was defined as the difference between a participant’s chronological age and age at menarche.

*Maternal body fat*. The percentage of maternal body fat was evaluated 30 days postpartum using bioelectrical impedance (InBody® 370, InBody CO. Seoul, Korea).

*Predicted height according to the height of parents*. At the participant’s first consultation, her father’s and mother’s height were also measured using a stadiometer (SECA model: 274, with precision 0.10 cm). These measurements were used to calculate the proportion of the adolescent’s projected height that she had reached. Participants who had reached 100% of maternal height and ≥92% of paternal height were considered to have reached full growth [5].

*Weight and height of the newborn*. Newborns’ weight (g), length (cm), and gestational age (weeks) were obtained from medical records.

#### Leptin, IGF-I, and estradiol processing

During gestational weeks 28, 32, and 36, 5 mL of blood samples were collected between 7 to 8 AM, after an 8–10 hour fast. Serum samples were frozen at -70°C until processing. We calculated the mean of the three measurement times of each hormone—leptin, IGF-I, and estradiol—and used the mean to represent the level of hormones during the third trimester of pregnancy. Leptin was measured with an ELISA technique using an absorbance microplate reader (ELISA Bio-Rad, model 680 Benchmark Plus, Bio-Rad, Hercules, CA, USA) and the ELISA Human Leptin Immunoassay kit (Quantikine® ELISA Human Leptin Immunoassay, R&D Systems Inc., Minneapolis, MN, USA), following manufacturer’s instructions. The range of the assay was between 1.0 and 100 ng/mL, with a sensitivity of 0.5 ng/mL. The experimental variation and the intra-assay variation coefficient were 2.0–6.0% and 2.0–5.0%, respectively. IGF-I was processed using a two-step sandwich immunoassay amplified by enzymes using the IGF-I ELISA kit (ADSL-10-2800 ACTIVE). The sensitivity was 0.01 ng/mL, and the coefficient of variation was <5%. Estradiol (estradiol-17 beta, E2) serum concentration was calculated using Immulite-1000 estradiol assay (Siemens Healthcare). The detection range was from 20 to 2000 pg/mL, the sensitivity was 15 pg/mL, and the variation coefficient was <5%.

#### Socioeconomic level

Information on participants’ socioeconomic level was determined through an interview with one of the parents or the guardian of each adolescent. The participant’s family was classified as a household, using the criteria set by the Mexican Association of Market Research and Public Opinion [32]. The categories used for this study were middle, low, and very-low class.

#### Ethical and research considerations

The Institutional Review Board and Ethics Committees from Instituto Nacional de Perinatología approved the study with registration number 212250–49451. Data gathering was confidential, taking ethical questions such autonomy and security into account. The guidelines of The Helsinki Declaration were followed. All pregnant adolescents received nutritional counseling to help them to adopt adequate eating habits. The recommendations provided to adolescents were per relevant official regulations defined by the Mexican Secretary of Health [33].

#### Statistical analyses

Our outcome variable was change in height, which was described first by calculating its mean and standard deviation. The dependent variable of change in height was evaluated in terms of different covariates. Covariates were categorized as follows: chronological age was classified into younger (≤15 y) and older adolescents (≥16 y) [34]. Participants’ height was classified in terms of their parent’s height [5]: either complete (≥92% of father’s height and ≥100 of mother’s height) or incomplete (<92% of father’s height and <100 of mother’s height). Percentage of body fat was divided into ≤29 and, ≥30% around our data’s median (30%); energy intake was classified into quartiles; gynecological age was classified according to the years after menarche (<4 years, exactly 4 years, and, ≥5 years). Breastfeeding was classified into participants who exclusively breastfed ≥6 months and those who breastfed for ≤5 months. We first compared the means of height change against these covariates using a one-way ANOVA test. We did not find any statistic differences in height change according to the covariates and therefore, do not present the post-hoc results in Table 1.

**Table 1:** Height gain (mm) by selected clinical, anthropometric, and sociodemographic variables.

	Mean ± SD	95% CI	p-value*
Energy intake (kcal)			0.234
≤1,700 (n = 42)	8.4±4	7.2–9.7	
1,701–2,000 (n = 42)	10.3±4.4	8.9–11.7	
2,001–2,300 (n = 42)	9.7±4.5	8.3–11.2	
≥2,300 (n = 42)	9.7±4.2	8.4–11.0	
Time spent exclusive breastfeeding			0.698
≥6 months (n = 76)	9.4±3.5	8.6–10.2	
≤5 months (n = 92)	9.6±4.8	8.6–10.7	
Age			0.442
11 to 15 years (n = 60)	9.2±4.3	8.1–10.3	
16 and 17 years (n = 108)	9.7±4.3	9.8–10.6	
BMI category			0.171
Normal (n = 138)	9.6±4.4	8.9–10.4	
Overweight (n = 23)	9.8±3.9	8.0–11.5	
Obesity (n = 7)	6.5±2.9	3.8–9.3	
Height in relation to paternal height (n = 127)			0.476
Incomplete (n = 63)	9.0±3.7	8.1–9.9	
Complete (n = 64)	9.5±4.3	8.4–10.0	
Height in relation to maternal height (n = 156)			0.838
Incomplete (n = 40)	9.4±3.9	8.2–10.7	
Complete (n = 116)	9.6±4.4	8.8–10.4	
Height in relation to parents’ height (n = 125)			0.880
Incomplete (n = 71)	9.3±3.6	8.4–10.1	
Complete (n = 54)	9.4±4.5	8.1–10.6	
Percentage of body fat			0.558
≤29% (n = 84)	9.4±4	8.5–10.1	
≥30% (n = 84)	9.7±5	8.7–10.8	
Bone age			0.110
Lower than chronological (n = 13)	11.1±3.5	9.0–13.2	
Equal to chronological (n = 71)	9.3±5.4	8.0–10	
Higher than chronological (n = 84)	8.1±5.6	6.8–9.3	
Gynecological age			0.918
0 to 3 years (n = 61)	9.3±4.7	8.2–10.6	
4 years (n = 40)	9.7±4.5	8.2–11.1	
5–8 years (n = 67)	9.6±3.8	8.7–10.6	
Socioeconomic level			0.466
Middle class (n = 50)	10±4.5	8.7–11.3	
Low class (n = 94)	9.5±4.3	8.6–10.4	
Very low class (n = 24)	8.6±3.8	7.0–10.3	

SD: Standard Deviation; CI: Confidence Interval; p: value by one-way ANOVA*

Change in height was compared with levels of leptin, IGF-I, and estradiol using a Person correlation coefficient. We also compared change in height in millimeters against the quartiles of serum levels of leptin (QI ≤9.5, QII 9.59–9.84, QIII 9.85–10.09, QIV ≥10.10), IGF-I (QI ≤155.5, QII 155.66–206.5, QIII 206.6–263.4, Q IV ≥263.5), and estradiol (QI ≤13214, QII 13215–14462, QIII 14463–16163, QIV ≥16164) using a one-way ANOVA.

We performed a Pearson χ2 test to compare height changes in tertiles and bone age in tertiles. To identify the possible variables associated with change in height, we performed a univariate general linear model to analyze the association between height gain and the continuous and categorical covariates. We utilized this model because it allowed us to adjust by potential confounders (bone age, and gynecological age, age at menarche, parents’ height, body fat, energy intake, and socioeconomic level). All data were analyzed using the 21^st^ version SPSS for Windows (IBM® Corp, North Castle, NY, USA). Statistical significance was declared at p <0.05.

## Results

One hundred and sixty-eight healthy pregnant teenagers participated in the study. Overall, pregnancies lasted an average of 39 ± 1 weeks, and babies were born with a mean weight of 3030 ± 378 g and a mean length of 49.5 ± 1 cm. There were 104 (62%) baby girls, and the rest were boys (38%). Participants ingested an average of 101 ± 25% of their recommended daily caloric intake. Of this total energy intake, an average of 49.9 ± 9% of the calories came from carbohydrates, 15 ± 3% from protein, and 34 ± 9% from fats. Between gestational week 28 and one year postpartum, the adolescent mothers had a mean increase in height of 9.5 ± 4 mm.

**Fig 2:**
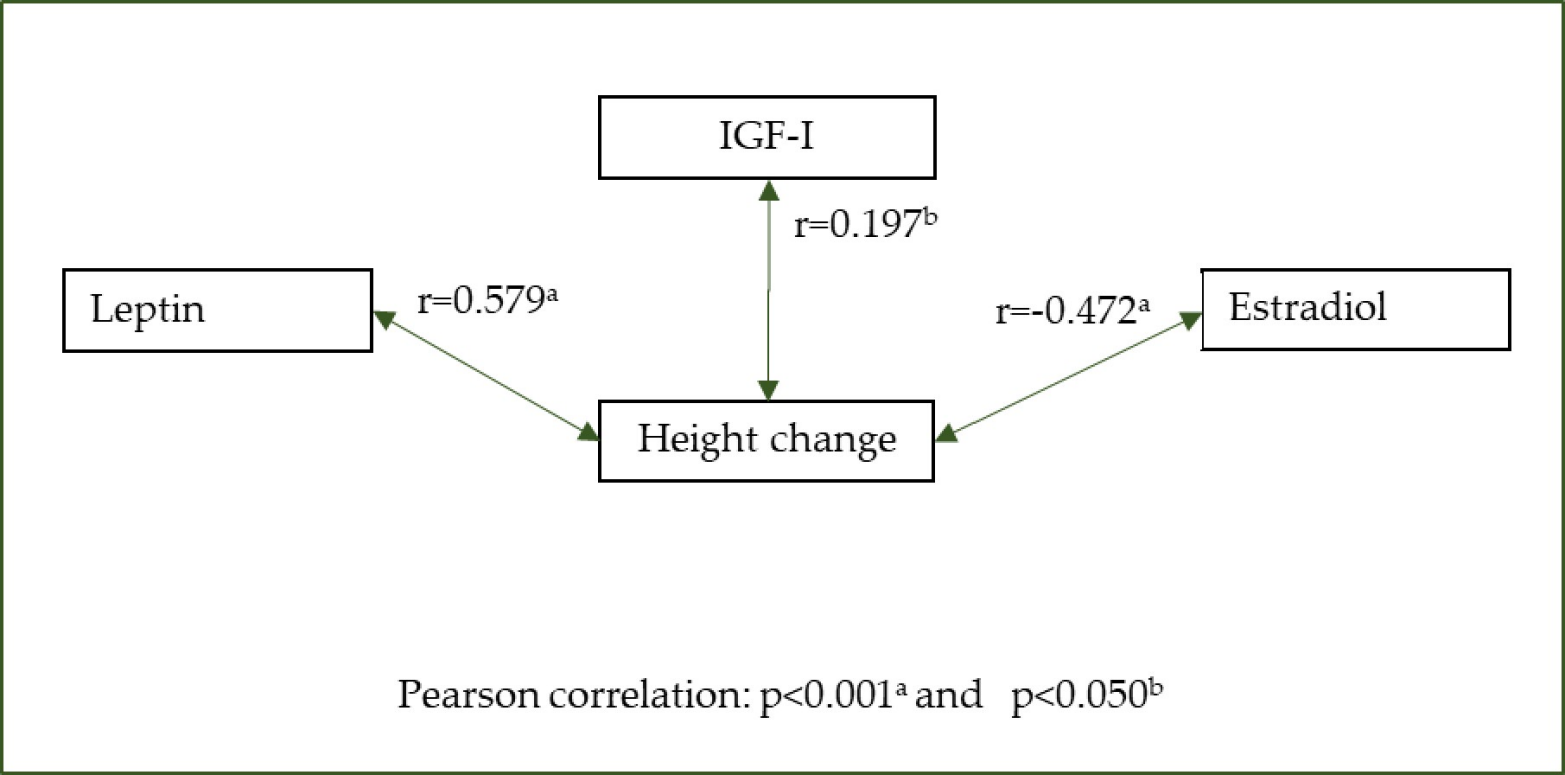
Pearson correlation between change in height and leptin, IGF-I, and estradiol.

Table 1 shows how participants’ change in height was similar across all the groups defined by anthropometric, dietary, and clinical variables. Serum concentrations of leptin in the third trimester was 20 ± 6.9 ng/mL, IGF-I was 214 ± 70 ng/mL, and estradiol was 14,603 ± 2,305 pg/mL. Change in stature between the 28^th^ week of gestation and 12 months postpartum was significantly correlated with leptin, IGF-I, and estradiol levels in the third trimester, as shown in (Fig 2).

**Fig 3:**
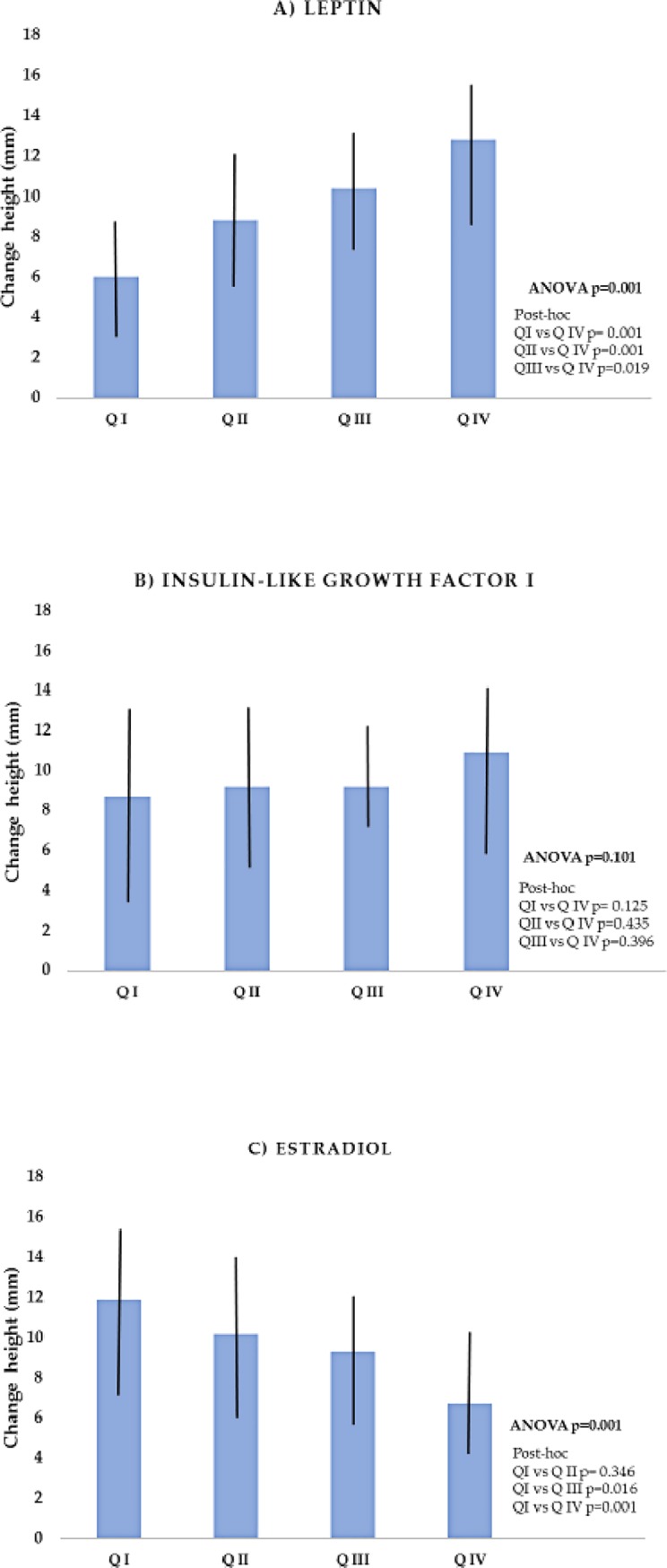
Change in height by quartiles of leptin (A), IGF-I (B) and, estradiol (C). ANOVA, Post-hoc by Bonferroni.

When we compared the change in height with the quartiles of the hormones, we observed that only leptin and estradiol showed statistically significant differences between quartiles. As stature increased, concentrations of leptin increased and estradiol decreased (Fig 3).

**Fig 4:**
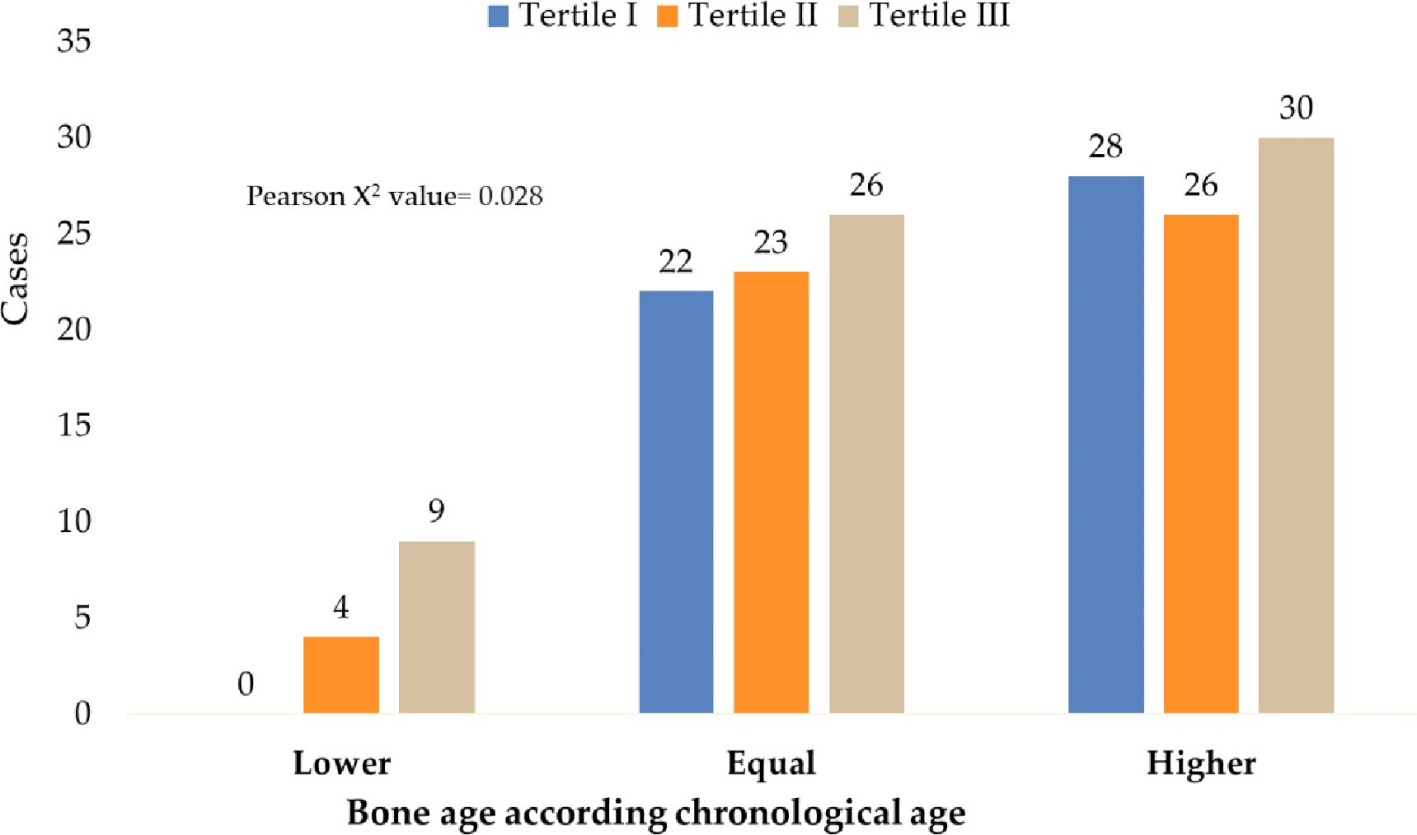
Bone age classification compared to chronological age, by tertiles of height increase.

Fig 4 shows how most participants with low bone age were in the tertile of highest growth (p = 0.028).

After describing the variables’ characteristics and performing bivariate analysis, the univariate model was performed (Table 2). The univariate general linear model showed that only leptin and estradiol from the third trimester of pregnancy were associated with the change in height (model’s R^2^ = 0.42). Concentrations of leptin explained 28.5% of height change while estradiol explained 11.1%. Nevertheless, IGF-I, chronological age, and breastfeeding had no association with height increase.

**Table 2:** Univariate general linear model to determine which variables predict an increase in height.

Covariate	B	Standard error	CI 95%	p-value	Eta squared partial
Breastfeeding (months)	0.004	0.006	-0.007, 0.016	0.473	0.003
Chronological age (years)	-0.031	0.024	-0.078, 0.015	0.188	0.011
IGF-I (ng/mL)	-0.011	0.078	-0.164, 0.143	0.892	0.016
Estradiol (pg/mL)	-0.013	0.003	-0.018, -0.007	0.001	0.111
Leptin (ng/mL)	0.652	0.081	0.492, 0.813	0.001	0.285

Adjusted by energy intake, parents’ height, body fat, bone age, gynecological age, and BMI. R^2^ = 0.44; Adjusted R^2^ = 0.42.

## Discussion

In this study, a sample of 168 pregnant adolescents grew a mean of 9.5 mm between the 28^th^ week of gestation and 12 months postpartum. WHO reference standards establish that people under 19 years of age should grow an average of 20 mm per year [35]. In addition to our participants having growth below the WHO reference, our participants had growth lower than non-pregnant adolescents from Colombia [36]. In this sense, it is essential to emphasize that although our participants were healthy, living in an urban residence and had prenatal medical care, the predicted height according to international standards was not reached, even considering the chronological age. Studies, such as that done by Zong et al. [37], have shown that an increased production of sex hormones during puberty causes morphological changes, which then leads to associated modification of anthropometric measures. Changes associated with pregnancy can interact and even counteract body morphology and growth processes induced by endocrine transformation in puberty.

During the last 100 years, the average height in the Mexican female population has remained among the ten lowest in Latin America [38]. In addition, Mexico is among the top three countries with the highest proportional frequency of teenage pregnancy in the Americas region [39] and is in the first place of teenage pregnancies in the Organization for Economic Cooperation and Development (OECD) member countries [40]. Taking these facts and the results of our research into account, we posit that adolescent pregnancy may be limiting the populational growth of Mexican adolescents. However, this is merely a preliminary hypothesis that should be explored through future research, because there are several factors associated, for instance: physical activity, diet, health status, and even family income and access to health services.

### Bone age, stature, and leptin, IGF-I, and estradiol levels

Comparing the bone age against chronological age allowed us to identify adolescents whose growth plates had not yet closed but did not reach levels WHO reference standards [35]. In addition, adolescents who had a bone age older than their chronological age were those who had the lowest height increase, as well as had higher concentrations of estradiol and lower leptin. However, the effect of bone age on growth was null in the univariate model.

In the present study on pregnant adolescents, there was no association between IGF-I and height gain. We found a weak correlation between IGF-I concentrations and change in height, but this association then disappeared after adjustment by potential confounding variables. In their third trimester of pregnancy, our participants had much lower mean levels of IGF-1 than reference adolescents from four different populations [41–44]. In fact, during their third trimester of pregnancy, our participants had a median level of IGF-1 corresponding to ranges for preteen girls (10 to 14 years old) in a Spanish population [41]. We hypothesize that these low concentrations of IGF-1 in our patients could be associated with the limited height increase in our sample. Nevertheless, this postulate should be examined through further research.

In our study, concentrations of leptin and estradiol explained 39.6% of the variability in the increase in height. Estradiol increases sharply during pregnancy, and increased estradiol concentrations are associated with higher probabilities of closure of growth plates [45]. As demonstrated in our study, pregnant adolescents could still grow, but probably to a lesser degree. Growth plates close approximately a decade after a teenager reaches her maximum height, meaning that a pregnant adolescent could still have time to grow [46]. We believe that this was the case in our participants, especially those who showed high concentrations of leptin. Leptin is an anabolic hormone that contributes to growth, and indeed, our model showed that 28.5% of the increased height was associated with leptin concentrations. The concentrations of leptin in the pregnant adolescents that we studied probably allowed them to grow, albeit below international growth standards [35]. This is consistent with information reported by Stevens-Simon et al. [47]. In their third trimester, our participants had leptin concentrations were higher than non-pregnant adolescents [48,49] but much less than pregnant women in the third trimester [50].

The vast majority of pregnant adolescents we studied did grow, but less than the WHO’s international growth references for adolescent girls [39]. The adolescents who grew less or did not grow were those who had the lowest serum concentrations of leptin. In contrast to previous reports, we observed that participants who grew less and had the lowest concentrations of leptin had a similar diet and nutritional status compared to those that grew more and had the highest concentrations of leptin. The explanation for this phenomenon could be found in other variables that were not evaluated in this investigation, for example, adiposity, ethnical characteristics, and inter-generational patterns of growth [38]. Those adolescents who showed the highest concentrations of leptin were also those with the greatest increase in height. This finding can also be explained by leptin’s anabolic effect and its effect on the lengthening of long bones [20,51]. This is consistent with the results of Scholl et al., who reported that higher concentrations of leptin during the third trimester of pregnancy were correlated with a greater increase in knee height [9]. In contrast, high estradiol concentrations were associated with a lower increase in height [46].

Our univariate linear model did not show differences in height increase between those who breastfed and those who did not. Our results are different from those reported by Rah et al., who observed that teenage girls who breastfed did not grow and lost weight while breastfeeding [52]. In addition, if we compare the amount of maternal body fat, our participants had a higher percentage of fat mass than the group of teenage mothers studied by Rah et al. (29 vs. 18%, respectively) [52]. This increased body fat mass could explain the limited but positive growth observed in our participants. Our participants’ greater body fat reserve could help them cover the necessary energy for breastfeeding and also, therefore, increase their height. Our patient sample also had appropriate caloric intake for their age and levels of physical activities and in accordance with international recommendations [53,54]. Therefore, we posit that breastfeeding probably does not affect adolescents’ growth as long as they have adequate energy intake.

In our study, we observed that breastfeeding, chronological age, and caloric intake were not associated with increased height. Indeed, despite adequate energy consumption and prenatal care in a tertiary care hospital, our patients did not grow within the WHO recommended range of growth for adolescent women. Therefore, the hypothesis arises that pregnancy could be a risk factor associated with interrupted adolescent growth; this topic will undoubtedly be a future area of research for our group.

### Strengths and limitations

The main strength of the present investigation is that it is a cohort design with a follow-up of 15 months in participants from an urban environment who have adequate energy intake and prenatal medical care. As far as we know, this is the first study that quantifies exactly how much these hormones contribute, in quantitative terms, to growth in pregnant Mexican adolescents. The analysis of covariates and confounding factors also contributes to the literature on teenage pregnancies, even because it is also about providing evidence about null relationships between certain variables

One of the limitations of the study is that IGF-I, leptin, and estradiol were only evaluated during pregnancy and not after birth; however, our study group was followed up closely for 12 months, gathering changes in other important variables. Another limitation is that we did not have a control group of non-pregnant adolescents to be able to argue that pregnancy during adolescence is a factor of risk that limits the growth of adolescent girls. To control for this, we compared our population’s growth against the WHO growth charts for adolescent girls, and in fact, found a statistically significant difference. This, therefore, allows us to compare our hospital population against international standards. Another limitation is that this study was only designed to be representative of adolescent mothers who receive medical care in our institution, which is not representative of the general population of Mexico City or indeed of Mexico. As INPer is tertiary care institution; our results may be different from adolescents who received care at primary care clinics or other hospitals. Nevertheless, we believe that ensuring uniform appropriate prenatal care and studying adolescents with adequate nutrient intake served to control those factors that may play an important role in growth and pregnancy. Therefore, this only heightens the strength of our findings that pregnancy is indeed a factor associated with lower increase in height in adolescent mothers.

## Conclusions

A population of pregnant adolescents in Mexico City gained, on average, 9.5 mm of height during pregnancy and one year after giving birth. This height gain is less than the average increase in height expected for non-pregnant adolescents according to WHO international growth standards. In these pregnant adolescents, serum concentrations of leptin and estradiol were associated with higher and lower height gain, respectively. Adolescents with the greatest increase in height were those with the highest concentration of leptin and IGF-I. This was in contrast with adolescents with the highest concentrations of estradiol, who had the lowest increase in height. Chronological age, gynecological age, and breastfeeding were not associated with the increase in height. The results of this study can be used to better understand the increase in height in adolescents during and after pregnancy, especially in populations with a high prevalence of teenage pregnancy.

We consider that our finding is not merely that leptin and estradiol are associated with the length growth, but rather the role that all the studied hormones play (or not) in growth.

## Abbreviation

IGF-I: Insulin-like Growth Factor I
BMI: Body Mass Index
INPer: Instituto Nacional de Perinatología
WHO: World Health Organization
